# Immobilization of Streptavidin on a Plasmonic Au-TiO_2_ Thin Film towards an LSPR Biosensing Platform

**DOI:** 10.3390/nano12091526

**Published:** 2022-05-01

**Authors:** Patrícia Pereira-Silva, Diana I. Meira, Augusto Costa-Barbosa, Diogo Costa, Marco S. Rodrigues, Joel Borges, Ana V. Machado, Albano Cavaleiro, Paula Sampaio, Filipe Vaz

**Affiliations:** 1 Centre of Molecular and Environmental Biology (CBMA), Department of Biology, University of Minho, 4710-057 Braga, Portugal; patricialexandra11@gmail.com (P.P.-S.); augusto.ac.barbosa@gmail.com (A.C.-B.); diogo.eccosta@gmail.com (D.C.); psampaio@bio.uminho.pt (P.S.); 2 Institute of Science and Innovation for Bio-Sustainability (IB-S), University of Minho, 4710-057 Braga, Portugal; 3 Physics Center of Minho and Porto Universities (CF-UM-UP), Campus de Azurém, University of Minho, 4800-058 Guimarães, Portugal; dianaisabelameira@gmail.com (D.I.M.); marcopsr@gmail.com (M.S.R.); fvaz@fisica.uminho.pt (F.V.); 4 Institute of Polymers and Composites (IPC) and Institute of Nanostructures, Nanomodelling and Nanofabrication (I3N), Campus de Azurém, University of Minho, 4800-058 Guimarães, Portugal; avm@dep.uminho.pt; 5 Laboratory of Tests, Wear and Materials, IPN-LED & MAT—Instituto Pedro Nunes, Rua Pedro Nunes, 3030-199 Coimbra, Portugal; albano.cavaleiro@dem.uc.pt; 6 Department of Mechanical Engineering, CEMMPRE—Centre for Mechanical Engineering Materials and Processes, University of Coimbra, Rua Luís Reis Santos, 3030-788 Coimbra, Portugal

**Keywords:** Au-TiO_2_ thin film, plasmonic biosensor, streptavidin–biotin, LSPR detection

## Abstract

Optical biosensors based on localized surface plasmon resonance (LSPR) are the future of label-free detection methods. This work reports the development of plasmonic thin films, containing Au nanoparticles dispersed in a TiO_2_ matrix, as platforms for LSPR biosensors. Post-deposition treatments were employed, namely annealing at 400 °C, to develop an LSPR band, and Ar plasma, to improve the sensitivity of the Au-TiO_2_ thin film. Streptavidin and biotin conjugated with horseradish peroxidase (HRP) were chosen as the model receptor–analyte, to prove the efficiency of the immobilization method and to demonstrate the potential of the LSPR-based biosensor. The Au-TiO_2_ thin films were activated with O_2_ plasma, to promote the streptavidin immobilization as a biorecognition element, by increasing the surface hydrophilicity (contact angle drop to 7°). The interaction between biotin and the immobilized streptavidin was confirmed by the detection of HRP activity (average absorbance 1.9 ± 0.6), following a protocol based on enzyme-linked immunosorbent assay (ELISA). Furthermore, an LSPR wavelength shift was detectable (0.8 ± 0.1 nm), resulting from a plasmonic thin-film platform with a refractive index sensitivity estimated to be 33 nm/RIU. The detection of the analyte using these two different methods proves that the functionalization protocol was successful and the Au-TiO_2_ thin films have the potential to be used as an LSPR platform for label-free biosensors.

## 1. Introduction

Biosensors have been widely developed as a tool for the environmental, food, and medical areas [1,2,3]. Due to their easy, rapid, low-cost, highly sensitive, and selective analysis, biosensors have a huge impact on the evolution of detection and diagnosis methods [4]. Usually, biosensor devices are composed of biorecognition elements (e.g., DNA, enzymes, and antibodies), for selective/specific binding with target analytes, and a transducer component converting the binding event into a readable signal. Based on the type of transducer, biosensors can be classified as electrical, thermal, and optical, among others [5].

Optical biosensors based on localized surface plasmon resonance (LSPR) are mentioned as one of the next generation of plasmonic label-free detection methods [6]. The optical phenomenon of LSPR occurs when an incident electromagnetic field interacts with noble metal nanoparticles (e.g., silver or gold), which can be dispersed in a dielectric matrix as a thin film [7,8,9]. As a result, the conduction band electrons of the nanoparticles oscillate in resonance with the incident electromagnetic wave, and this interaction enhances the local electromagnetic field and leads to a strong absorption band [10,11,12]. The LSPR properties are influenced by several factors, such as the surrounding dielectric matrix, the nanoparticles’ features (concentration, size, shape, and distribution), and the refractive index of the environment [13,14,15]. Therefore, it is possible to tune the LSPR response of nanocomposite thin films to be sensitive to subtle refractive index changes [16,17,18], such as those induced by the molecular interactions near the nanoparticles’ surface, providing effective transduction of the binding events into an LSPR band shift [19,20]. So, no conjugating reporter molecules are required, making LSPR-based sensing label-free, portable, and low-cost and allowing real-time analysis [21]. Moreover, the optical hardware is smaller, more affordable, and simpler compared to other optical sensors [22].

Many efforts have been made to develop LSPR-based sensors using different approaches such as (1) solution phase-based colloidal nanoparticles, (2) nanoparticle-coated optical fiber platforms, and (3) flat substrate-based platforms [23]. Concerning the last approach, plasmonic thin films are promising for sensing platforms, namely magnetron-sputtered nanocomposite thin films containing noble metal nanoparticles (Au and/or Ag) dispersed in dielectric or semiconducting matrixes [24,25,26]. As solid supports, the nanoparticles are immobile in an inert solid matrix, preventing nanoparticle aggregation, a highly observed nuisance in colloidal solutions [27]. Moreover, flat substrate-based LSPR sensors show higher reproducibility, signal-to-noise ratio, and sensitivity compared to sensing approaches based on isolated nanoparticles [28,29,30,31]. Through adjustments in the deposition parameters and post-deposition treatments, it is possible to modify the plasmonic thin films’ nanostructure to be used as both platforms and transducers, detecting and converting the molecular interactions into an LSPR band shift [32,33,34,35].

Biotin (or vitamin B7/vitamin H) is a small molecule that binds with high affinity, specificity, and stability to streptavidin, a tetrameric protein with four identical binding sites for biotin [36,37]. The femtomolar affinity of biotin to streptavidin is one of the highest reported for a non-covalent interaction [38], and therefore it is commonly used as a model system for evaluating the performance of a biosensor [39,40,41]. Sensors can be coated with streptavidin protein, and biotin can be functionalized with any molecule, including enzymes such as horseradish peroxidase (HRP). The strong, specific, and stable bond between streptavidin and biotin can be easily confirmed by HRP activity [42].

Considering the demand for sensitive and fast biosensors, the development of plasmonic thin films as sensing platforms is beneficial. Therefore, in this work, the biosensing ability of the nanoplasmonic thin films, composed of gold nanoparticles dispersed in a titanium dioxide matrix (Au-TiO_2_), was tested through the attachment of molecular biorecognition layers. These thin films were produced by reactive direct current (DC) magnetron sputtering and subjected to post-deposition treatments to promote the growth of the Au nanoparticles throughout the TiO_2_ matrix [24]. Streptavidin protein was immobilized on the surface of the thin films, as a biorecognition layer, followed by binding with biotin conjugated with HRP, in a procedure similar to enzyme-linked immunosorbent assay (ELISA) [43]. Then, the plasmonic platform’s ability to detect the analyte (biotin molecule) was evaluated through two different methods: the detection of HRP activity and the LSPR band shift. This work aimed to develop an LSPR biosensor based on plasmonic Au-TiO_2_ thin films, as well as to demonstrate the efficiency of the immobilization protocol, using the streptavidin and biotin–HRP interactions.

## 2. Materials and Methods

### 2.1. Preparation and Characterization of the LSPR Biosensor Platform

#### 2.1.1. Deposition of Au-TiO_2_ by Reactive DC Magnetron Sputtering

Thin films, composed of gold (Au) dispersed in a titanium dioxide (TiO_2_) matrix, were deposited by one-step reactive DC magnetron sputtering in a custom-made high-vacuum chamber. Two different types of substrates were used for the depositions, namely glass lamellae (ISO8037) and monocrystalline silicon (Si) wafers with (100) orientation. Before the depositions, these substrates were cleaned with oxygen (O_2_) plasma, at a partial pressure of 60 Pa for 5 min, to remove impurities from the substrate’s surface. Then, to activate the substrate’s surface and improve the thin film’s adhesion, argon (Ar) plasma was applied at a partial pressure of 60 Pa for 15 min. Those plasma treatments were performed at a base pressure of 20 Pa in a Diener (Zepto Type Model) instrument, operating at a power of 50 W, using a 13.56 MHz RF generator.

A high purity (99.99%) and rectangular (200 nm × 100 nm × 6 mm) titanium target was used as a cathode for the production of thin films. Gold pellets with a total surface area of 16 mm^2^ and a thickness of 0.5 mm were placed symmetrically in the preferential erosion zone of the target for the homogeneous incorporation of the noble metal in the TiO_2_ matrix. The substrates were placed on a grounded rotating substrate holder, with a constant rotation speed of 12 rpm.

Before the depositions, the sputtering chamber was pumped down to 3.2 × 10^−4^ Pa. The deposition of the thin films occurred at a working pressure of 4.0 × 10^−1^ Pa, using a plasma of Ar (working gas) and O_2_ (reactive gas). The Ar and O_2_ flows were kept constant during the deposition at partial pressures of 3.6 × 10^−1^ Pa and 4.0 × 10^−2^ Pa, respectively. The deposition time was 30 min, based on previous work [24]. The applied direct current density was 100 A m^−2^, and the (negative) target potential was 400 V.

#### 2.1.2. Post-Deposition Annealing Treatment to Promote the Growth of the Au Nanoparticles

Au-TiO_2_ thin films were subjected to an in-air annealing treatment to promote the diffusion of Au atoms and hence the growth of Au nanoparticles throughout the matrix, enhancing the LSPR response, as reported in other works [14,44,45]. The thermal treatments were performed in a programmable furnace (Termolab FP21) at 400 °C. The chosen annealing temperature was reached by raising the temperature at a rate of 5 °C/min until the desired temperature was attained, fixing this temperature for 5 h. The samples were allowed to cool freely inside the furnace before reaching room temperature.

#### 2.1.3. Surface Modification through Plasma Etching

The surface of the nanoplasmonic thin films was modified by plasma etching, using an Ar atmosphere at a partial pressure of 60 Pa for 60 min. The etching exposes the Au nanoparticles at the thin film’s surface, increasing the nanoparticles’ surface area available to the targeted biological analytes. This surface modification also increases the thin film’s sensitivity, as reported in previous work [24]. A Diener (Zepto Type Model) instrument, using a 13.56 MHz RF generator, was used for plasma etching, operating at a base pressure of 20 Pa and 50 W of power. Four independent samples were used to estimate (mean ± standard deviation) the effect of plasma treatment on the LSPR band.

#### 2.1.4. Characterization of Au-TiO_2_ Thin Films

The in-depth chemical composition profile in thin films deposited on silicon wafers was evaluated by Rutherford backscattering spectrometry (RBS) with a Van de Graff accelerator. The measurements were made in a small chamber, where three detectors were installed: a standard detector at 140° and two pin-diode detectors located symmetrically to each other, both at 165°. Spectra were collected for 2 MeV ^4^He^+^ beams at normal incidence (0°). The obtained RBS data were analyzed with IBA DataFurnace NDF v10.0b [46], and the double scattering and pileup were calculated with the algorithms given in N.P. Barradas et al. (2005) [47] and N.P. Barradas et al. (2006) [48], respectively.

The thickness of the thin films, before and after Ar plasma treatment, was evaluated in cross-sections of thin films deposited on silicon wafers. A scanning electron microscope, NanoSEM—FEI Nova 200 (FEG/SEM), operating at 10 keV was used.

#### 2.1.5. Modification and Measurement of the Thin Films’ Hydrophilic Property

The O_2_ plasma treatment has been reported as a protein immobilization promoter [49]. Therefore, the surface hydrophilic property of the Au-TiO_2_ nanoplasmonic thin film was modified by applying an O_2_ plasma at a partial pressure of 60 Pa and 37.5 W power. The exposure times were 10, 30, 60, 120, 150, 180, 210, and 300 s to evaluate the influence of a reactive plasma on thin films’ surface properties. The base pressure was 20 Pa in the Diener (Zepto Type Model) instrument, using a 13.56 MHz RF generator. Three independent samples were used to estimate (mean ± standard deviation) the effect of plasma treatment on optical properties.

The thin films’ wettability was characterized by contact angle measurements, as a function of the time of exposure to O_2_ plasma. The contact angle measurements were performed using the sessile drop in static mode, at room temperature and humidity in an OCA 20 instrument with a CCD video camera (resolution of 752 × 582 pixels) from Dataphysics Instruments GmbH (Filderstadt, Germany). Deionized water (3 µL drop) was used as the test liquid. The contact angle was calculated according to the Young–Laplace method [50], using the software SCA 20. At least 5 measurements were performed for each condition, using 3 samples, and the average contact angle was calculated. The contact angle values were plotted and analyzed using one phase decay in GraphPad Prism 9 software.

### 2.2. Protein Immobilization and Binding Assay

An in-house protocol built on standard HRP-based colorimetric ELISA protocol [43] was used for the detection of proteins’ immobilization on thin-film surfaces. The streptavidin–biotin pair was used as molecules for receptor–ligand binding, in which biotin is conjugated with HRP.

#### 2.2.1. Concentration Optimization

First, the ideal concentrations of streptavidin–biotin to be used were determined. So, a matrix of different concentrations of streptavidin (Millipore, Burlington, MA, USA) and biotin–HRP (Thermo Scientific, Waltham, MA, USA) ranging from 0.0025 to 25 µg/mL and from 0.0075 to 75 µg/mL, respectively, were used in the assay, carried out in 96-well plates (Thermo Scientific, Waltham, MA, USA).

Streptavidin was incubated for 1 h in each well, followed by washing steps with phosphate-buffered saline (PBS, pH 7.4), with 0.05% Tween-20 (PBS-T, from Sigma, St. Louis, MO, USA). To block unspecific binding sites, 2% bovine serum albumin (BSA, from Sigma-Aldrich, St. Louis, MO, USA) in PBS-T was added for another hour and then washed with PBS-T. The conjugated ligand, biotin–HRP, was incubated for 30 min and then washed with PBS-T. The substrate 3,3′,5,5′-tetramethylbenzidine (TMB, from Sigma-Aldrich, St. Louis, MO, USA) was added to the wells, to be converted into a colored product by the enzyme HRP. The enzymatic reaction lasted for 12 min and was stopped by adding phosphoric acid (H_3_PO_4_ 1 M, from PanReac AppliChem, Darmstadt, Germany). The absorbance of the reaction product was measured at 450 nm, using a SPECTRAmax Plus 384 (Molecular Devices, San Jose, CA, USA). Samples with no streptavidin or biotin–HRP (blank) and samples with no specific binding (containing either only streptavidin or biotin–HRP) were used as controls. The optimal streptavidin–biotin ratio was calculated from the absorbance results.

#### 2.2.2. Influence of O_2_ Plasma on Streptavidin Immobilization

Kim et al. reported the enhancement of proteins’ immobilization on surfaces after O_2_ plasma treatment [49]. Therefore, an O_2_ plasma was applied for 60 s to Au-TiO_2_ thin films, at a partial pressure of 60 Pa and 37.5 W power.

Then, a matrix of different concentrations of streptavidin and biotin–HRP, ranging from 1.25 to 25 µg/mL and from 0.375 to 1.5 µg/mL, respectively, were used in the assay. The Au-TiO_2_ thin films were incubated for 1 h with streptavidin, and the procedure followed was the same as the experimental protocol described in Section 2.2.1. Glass substrates previously treated with O_2_ plasma were used as immobilization control, and Au-TiO_2_ thin films and glass substrates without previous O_2_ plasma treatment were used to assess the effect of O_2_ plasma on streptavidin immobilization. Blank sample and non-specific binding (containing either only streptavidin or biotin–HRP) samples were also used as binding controls.

#### 2.2.3. Streptavidin Immobilization on Thin Film’s Surface

Based on the ideal ligand–receptor ratio and surface binding area, an extrapolation was made for protein immobilization on the thin film’s surface. To enhance protein immobilization, the Au-TiO_2_ thin film’s surface was previously activated with O_2_ plasma treatment (60 s and 37.5 W). Then, streptavidin was immobilized for 1 h, and the procedure followed was the same as the one mentioned above in Section 2.2.1. Nine independent thin films and 9 glass substrates (as immobilization control) were used to assess the repeatability. Blank sample and non-specific binding (containing either only streptavidin or biotin–HRP) samples were also used as binding controls.

### 2.3. Optical Response of the LSPR Biosensing Platform

The optical analysis of the Au-TiO_2_ thin films, namely the measurement of the LSPR band in transmittance mode, was performed in a custom-made optical system, with a light source (tungsten lamp), a custom sample holder, and an Ocean Optics HR4000 spectrometer, connected by optical fibers [51].

#### 2.3.1. Refractive Index Sensitivity of Au-TiO_2_ Thin Films

The refractive index sensitivity (RIS) of the produced Au-TiO_2_ thin films was determined by monitoring their optical transmittance while immersed in solutions with different refractive indexes. Sucrose solutions of 20% (*w*/*w*) (η = 1.3639 RIU), 30% (*w*/*w*) (η = 1.3812 RIU), 40% (*w*/*w*) (η = 1.3999 RIU), and 50% (*w*/*w*) (η = 1.4201 RIU) were used in the experiments. The transmittance spectra were monitored for 1 min (for each half-cycle), and each spectrum was acquired using an integration time of 4 ms and an average of 500 scans. Data were processed by the NANOPTICS software to identify changes in the T-LSPR wavelength peak [52]. RIS was calculated using the equation RIS = Δλ/Δη (nm/RIU), assuming a semi-infinite layer of the aimed solution, where Δλ represents the peak wavelength shift and Δη the difference of the refractive index of surrounding media [27].

#### 2.3.2. LSPR Response of the Streptavidin-Immobilized Thin Films to Biotin–HRP

The LSPR response of the streptavidin-immobilized Au-TiO_2_ thin films in the presence of the analyte was evaluated. This experimental step was crucial for assessing their potential as a biosensing platform. Therefore, the surface of the thin films (the LSPR platform) was immobilized with streptavidin and blocked with BSA as described above, followed by a 30 min incubation with PBS, to build the biosensor. The optical spectra were recorded, corresponding to the basal spectra of the biosensor. Finally, the LSPR biosensor was incubated for 30 min with the ideal concentration of biotin–HRP, and the optical response was measured. In each measurement, the LSPR biosensors were immersed in 1 mL of PBS and the optical spectra were monitored for 10 min in transmittance mode. An integration time of 4 ms and an average of 500 scans were used to acquire each spectrum. The repeatability parameter was determined in 3 independent measurements. Data were processed by the NANOPTICS software to assess the LSPR response of the biosensor, identifying changes in the T-LSPR band peak [52].

Finally, TMB was added to confirm the binding of biotin–HRP to streptavidin-immobilized thin films and for correlation with LSPR response. After 12 min incubation, a colored product was detected.

## 3. Results and Discussion

### 3.1. Characterization of Au-TiO^2^ Thin Films

#### 3.1.1. Chemical, Optical, and Morphological Properties of Au-TiO_2_ Thin Films

Materials’ properties affect biosensor development, so different characterization techniques were applied to analyze the microstructural characteristics and optical response of the plasmonic thin films. The atomic concentration (at. %) of each chemical element present in the thin film, after thermal treatment at 400 °C, was determined by RBS analysis. The annealed Au-TiO_2_ thin films are composed of 20.2 ± 0.5 at. % of gold. The O atomic concentration was found to be 50 ± 5 at. %, and the amount of Ti was found to be 30.4 ± 0.5 at. %. Nevertheless, given the uncertainties of the measurements, the matrix can be considered nearly stoichiometric.

**Figure 1:**
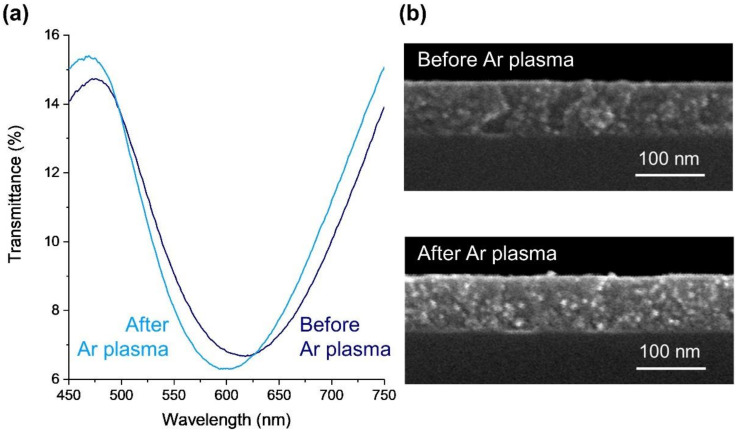
Effect of Ar plasma treatment on the optical and morphological properties of the Au-TiO_2_ thin films annealed at 400 °C: (**a**) optical transmittance spectra and (**b**) cross-section micrographs before and after Ar plasma.

Regarding the optical responses, Au-TiO_2_ thin films were evaluated by spectrophotometry in transmittance mode. The as-deposited film is transparent to the visible light and lacks an LSPR band, as the non-crystallized nanoparticles formed during the sputtering process are too small (typically below 10 nm) [53]. The LSPR band appears after annealing treatment at 400 °C due to the growth and coalescence of the Au nanoparticles dispersed throughout the TiO_2_ matrix, which typically crystallizes in the anatase phase, as previously observed [26,54,55,56]. The transmittance band minimum is positioned at a wavelength of 618 nm, corresponding to T = 6.7% (Figure 1a).

To develop nanoplasmonic thin films as a sensing platform, biorecognition elements must be immobilized on their surfaces to detect the analyte. Nevertheless, since the plasmon decay length of the nanoparticles is typically below a few tens of nanometers, and as demonstrated by discrete dipole approximation simulation models [57], the matrix where the nanoparticles are embedded might hinder the platform’s sensitivity. On the other hand, proteins can be immobilized as biorecognition elements by adsorption on the Au nanoparticles [58]. Due to these reasons, the Au nanoparticles embedded in the TiO_2_ matrix need to be partially exposed to the environment [27], allowing the immobilization of the molecular recognition elements. As shown in previous works [24,26], to enhance the density of gold nanoparticles at the film’s surface, an Ar plasma treatment was applied, giving rise to an LSPR band blueshift of about 16.3 nm ± 3.7 nm (Figure 1a). This shift is due to an increase in the number of nanoparticles exposed to air, which become partially embedded in the TiO_2_ matrix, thus decreasing the average refractive index surrounding them. However, as can be observed in Figure 1b, the plasma treatment did not cause a considerable etching of the matrix. In fact, in SEM micrographs, the annealed Au-TiO_2_ thin films presented an average thickness of 93 ± 1 nm, and after the Ar plasma treatment, they exhibited an average thickness of 94.8 ± 0.8 nm. This implies that the plasma etching probably removed only some weakly adhered layers and some chemical contaminants on the thin film’s surface (such as hydrocarbon layers) [24].

#### 3.1.2. Au-TiO_2_ Thin Films’ Hydrophilic Properties

The biosensor development is determined by the adhesion of the biorecognition layer to the platform’s surface. The presence of hydroxyl groups on the biosensor’s surface promotes the adsorption of biomolecules [49,59]. Therefore, the Au-TiO_2_ thin films were subjected to an activation plasma (O_2_ plasma) at different exposure times, and the effect was evaluated through wettability measurements, based on the contact angle of deionized water droplets. The contact angle is defined as the tangent of the liquid–vapor interface and the solid surface at the three-phase contact line [60]. Surfaces with contact angles lower than 65° are considered hydrophilic due to the presence of polar functional groups [61].

**Figure 2:**
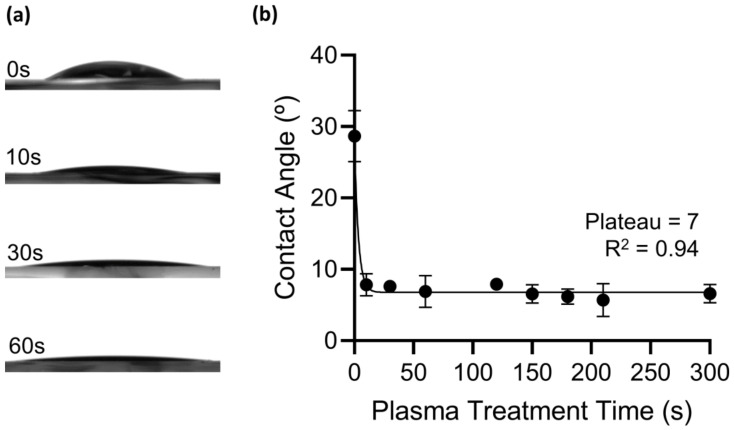
Hydrophilic properties of the Au-TiO_2_ thin films. (**a**) Photographs of a water droplet and the (**b**) contact angle (in degrees) measured as a function of O_2_ plasma treatment time in seconds (s) on the surface of Au-TiO_2_ thin films.

Without O_2_ plasma treatment (0 s), the Au-TiO_2_ thin film’s surface is already considered hydrophilic since it produced a contact angle of 29 ± 4° (Figure 2). After 10 s of O_2_ plasma treatment, the contact angle value significantly dropped to 8 ± 1°. Increasing the time of O_2_ plasma treatment did not induce major changes in contact angle value, and the plateau was reached after 60 s at 7°. From here, the thin films were treated with O_2_ plasma for 60 s before immobilization with the biorecognition layer.

**Figure 3:**
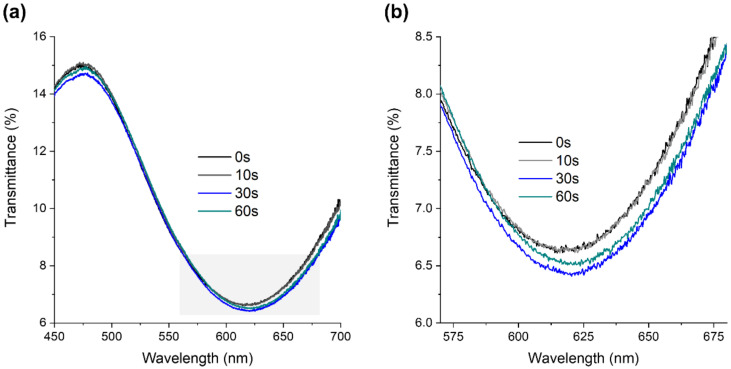
LSPR band evolution as a function of O_2_ plasma treatment time (0, 10, 30, and 60 s) of the Au-TiO_2_ thin film annealed at 400 °C: (**a**) transmittance spectra from 450 to 700 nm and (**b**) close-up of the highlighted area in graph a.

The effect of O_2_ plasma on the LSPR band was also evaluated, and it was observed that the exposure time of 60 s caused an LSPR band shift, to the right (redshift), of 4 ± 1 nm (Figure 3). Therefore, the O_2_ plasma changed the thin film surface wettability, increasing the hydrophilicity through the formation of hydroxyl groups, and induced minor changes in the LSPR band.

### 3.2. Streptavidin Immobilization and Binding Assay

The model receptor–analyte system, streptavidin–biotin, was chosen since it presents a high binding affinity between the molecules, and it is widely studied and used in biological fields [62]. Therefore, streptavidin was used as a biorecognition element to be immobilized on the surface of the Au-TiO_2_ thin films, and the analyte was biotin–HRP. The efficiency of the streptavidin–biotin binding at the biosensor’s surface is studied in this section.

**Figure 4:**
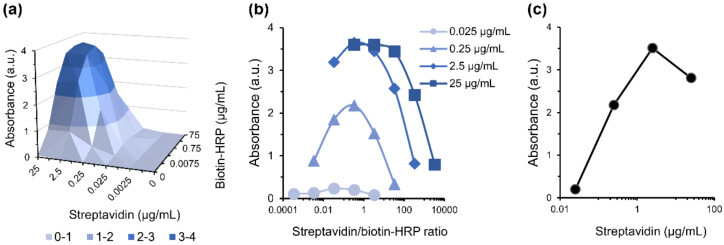
Streptavidin/biotin–HRP binding assay. (**a**) Absorbance values measured as a function of different streptavidin and biotin–HRP concentrations (µg/mL); (**b**) absorbance values measured as a function of streptavidin/biotin ratio, for streptavidin concentrations between 0.025 and 25 µg/mL; (**c**) absorbance values measured as a function of streptavidin concentration (µg/mL), for all combinations of streptavidin/biotin–HRP at the ratio of 0.33.

In a first approach, the concentrations of streptavidin and biotin–HRP were optimized. The streptavidin–biotin binding was determined by the absorbance of TMB oxidation by HRP, following a protocol based on ELISA in a 96-well plate. To evaluate the optimal streptavidin concentration on the well’s surface, different amounts were tested (from 0.0025 to 25 µg/mL) and conjugated with different amounts of biotin–HRP (from 0.0075 to 75 µg/mL). Absorbance data obtained were plotted as a function of streptavidin and biotin–HRP concentrations (Figure 4a). The results indicate that the increase in the amount of streptavidin had a greater impact on the absorbance values. The maximum absorbance (3.6) was achieved at 2.5 µg/mL of streptavidin, and raising its concentration leads to a reduction in the conjugate formation (Figure 4a).

The streptavidin/biotin–HRP ratios as a function of absorbance, for the tested streptavidin concentrations, were plotted to determine the optimal ratio, which was found to be 0.33 for every condition (Figure 4b). Plotting the absorbance values of 0.33 ratios as a function of streptavidin concentration, it was concluded that the ideal condition was 2.5 µg/mL of streptavidin with 7.5 µg/mL of biotin–HRP (Figure 4c). This value is very close to the expected 1:4 streptavidin/biotin known ratio; the difference could be related to streptavidin adsorption to the well’s surface, which prevents the binding of biotin to all the binding sites. Moreover, this could also be due to the conjugation of biotin with HRP, a large enzyme, which hinders the binding of other biotin molecules to the same streptavidin protein.

**Figure 5:**
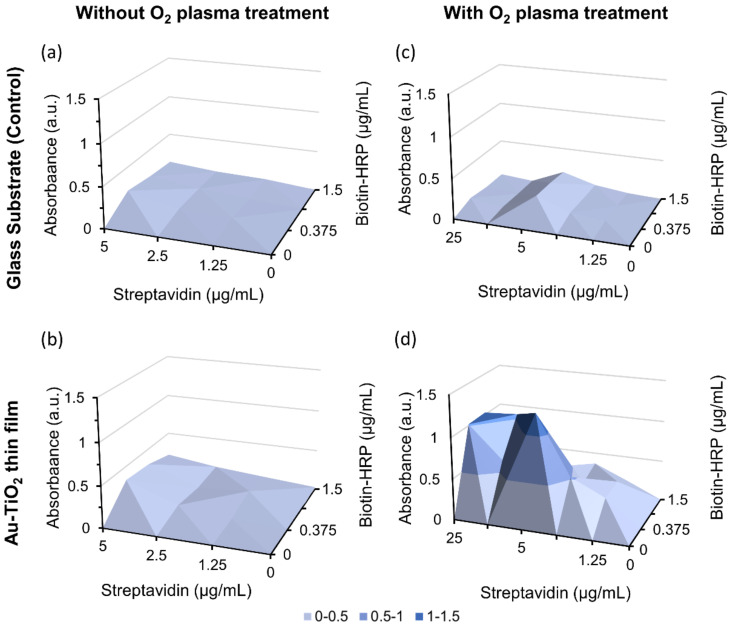
Influence of O_2_ plasma treatment on the immobilization of streptavidin on the thin film’s surface. Absorbance values measured as a function of streptavidin and biotin–HRP concentrations, without O_2_ plasma treatment in (**a**) glass substrate and (**b**) Au-TiO_2_ thin film and with O_2_ plasma treatment in (**c**) glass substrate and (**d**) Au-TiO_2_ thin film.

As previously shown, the O_2_ plasma treatment increased the hydrophilicity of the thin film’s surface. To evaluate the influence of O_2_ plasma treatment on protein immobilization and validate its importance in the adhesion of the biorecognition layers, O_2_ plasma treatment was applied on the glass substrates and Au-TiO_2_ thin films, followed by the incubation with streptavidin, ranging from 1.25 to 25 µg/mL, according to the described experimental protocol. Results show that the thin films without plasma treatment (Figure 5b) behave similar to glass substrates either with (Figure 5c) or without plasma treatment (Figure 5a). This demonstrates that streptavidin binds to the thin film’s surface, but only if treated with O_2_ plasma (Figure 5d). Furthermore, no signal was detected in the non-specific binding controls. Thus, all thin films must be subjected to O_2_ plasma treatment before the immobilization protocol.

Therefore, the immobilization protocol of the Au-TiO_2_ thin film’s surface was designed based on the aforementioned findings. Comparing the wells’ area (0.63 cm^2^) with the thin films’ surface area (1.125 cm^2^) and the optimal streptavidin/biotin–HRP ratio, an extrapolation of the protein concentration was made to achieve maximum binding. Hence, the determined ideal concentration of streptavidin to be immobilized on the Au-TiO_2_ surfaces was 4.5 µg/mL, and that of biotin–HRP was 13.5 µg/mL. After TMB oxidation by HRP, the average absorbance value in the immobilized Au-TiO_2_ thin films was 1.9 ± 0.6. As expected, no binding was observed with the glass substrate controls or with the non-specific binding control samples.

The results reveal the success of the streptavidin immobilization on the surface of the thin films and the efficient streptavidin–biotin binding. The described ELISA-based protocol resulted as being a proper method for immobilizing biorecognition elements in plasmonic thin-film surfaces towards the development of sensing platforms integrating LSPR transducers.

### 3.3. LSPR Biosensing Platform Sensitivity

#### 3.3.1. Refractive Index Sensitivity of the Au-TiO_2_ Thin Films

**Figure 6:**
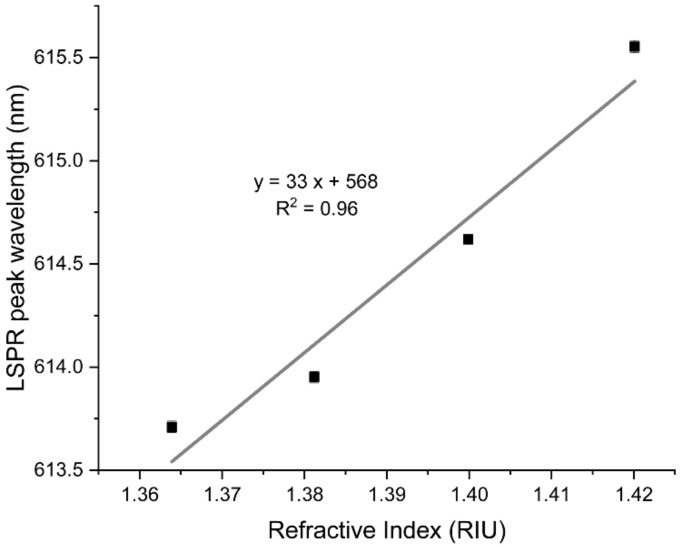
LSPR peak wavelength of the Au-TiO_2_ thin films as a function of the refractive index of the different sucrose solutions (20, 30, 40, and 50% (*w*/*w*)). The solid line is a linear fit to the data.

The sensing performance of the LSPR thin-film platforms is dependent on the ability to distinguish between different refractive indexes (η) of the surrounding media. Therefore, the transmittance spectra of the Au-TiO_2_ thin films were measured in the presence of sucrose solutions of four different concentrations with increasing refractive index values. Figure 6 plots the LSPR peak wavelength of the Au-TiO_2_ thin films as a function of the refractive index of the different sucrose solutions. As evidenced, increasing the refractive index of the surrounding medium resulted in an LSPR band redshift. The linear fit yielded a correlation coefficient R^2^ of 0.96 and a slope of 33 ± 5 nm/RIU, corresponding to the RIS, defined as the shift in LSPR wavelength per refractive index unit (RIU). However, the sensitivity of the film is still low when compared to the solution-based nanoplasmonic systems (≈200 nm/RIU) [63]. This might be related to the thickness of the film, which is about 95 nm, well above the plasmon decay length of the nanoparticles. Hence, the nanoparticles that effectively contribute to the detection signal are the ones closer to the surface of the film. Most of the nanoparticles are embedded in the matrix, so their signal contribution is defined by the dielectric matrix refractive index, and they do not “sense” the refractive index variations on the surface of the film [16,64]. Therefore, decreasing the film’s thickness towards a value comparable to the plasmon decay length should be considered to improve the sensitivity. Another possible way to optimize the sensitivity is to tune the nanoparticles’ features, such as the aspect ratio, concentration, and size distribution [65].

#### 3.3.2. LSPR Response of the Streptavidin-Immobilized Thin Films to Biotin–HRP

**Figure 7:**
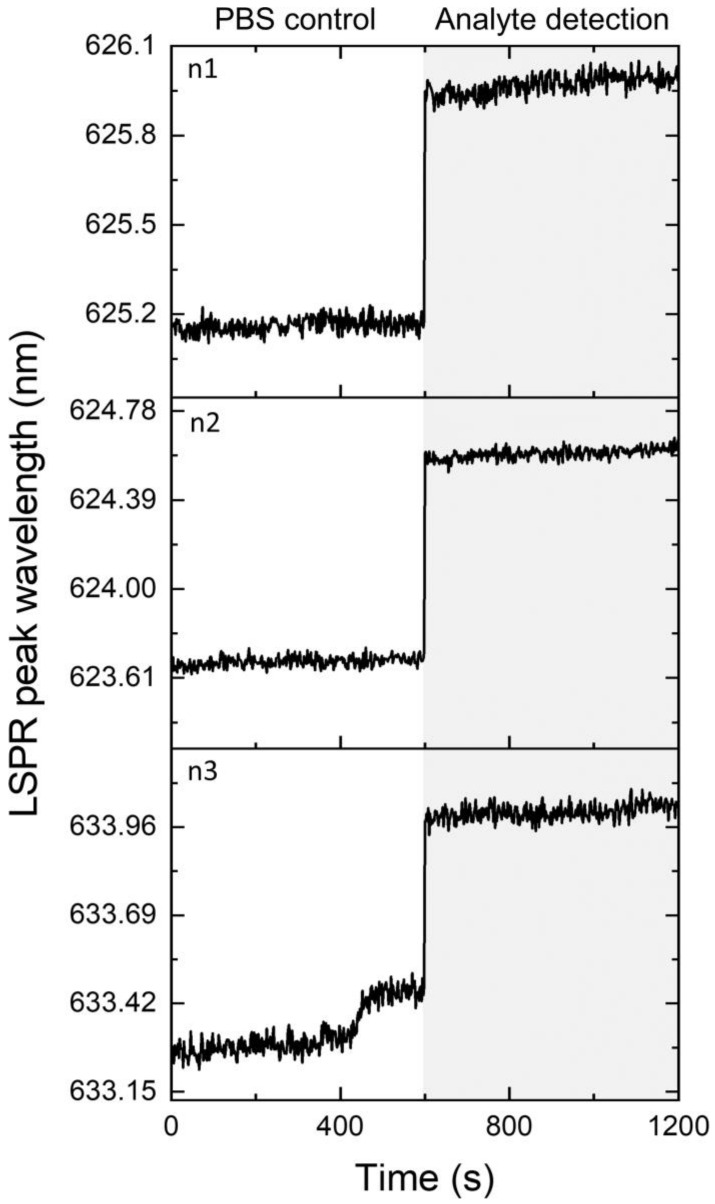
Ten-minute LSPR peak wavelength monitoring of the produced Au-TiO_2_ biosensor after incubation with PBS (control) and after the incubation with 13.5 µg/mL biotin–HRP (analyte detection). n1, n2, and n3 are the three independent samples tested.

The performance of streptavidin-immobilized Au-TiO_2_ thin films as sensing platforms was tested. The addition of the analyte (biotin–HRP) to the biosensor’s surface caused an LSPR band shift of 0.8 ± 0.1 nm towards a longer wavelength, as seen in Figure 7. This LSPR shift is related to an increase in the refractive index of the region surrounding the nanoparticles, induced by biotin molecules that were bound to the immobilized streptavidin. This relatively low LSPR band shift, compared to other reports [66], might be related to the poor sensing ability of the LSPR platform (RIS = 33 nm/RIU). In addition, different concentrations of biotin–HRP were added to the biosensor’s surface, but the low signal-to-noise ratio did not allow any measurable LSPR sensing response. Thus, the biosensor only presented an optical response in the presence of the ideal concentration of the analyte.

Additionally, to prove that this binding was specific, TMB was added to start the enzymatic reaction. The observed colored product proved the presence of biotin linked to streptavidin immobilized on the thin film’s surface. This detection method confirms that the functionalization protocol was successful and Au-TiO_2_ thin films immobilized with streptavidin can be used as LSPR biosensors. Despite this, additional optimization of the LSPR thin-film platform’s sensitivity should be envisaged.

## 4. Conclusions

In this work, an LSPR sensing platform based on streptavidin-immobilized Au-TiO_2_ thin films was developed. Nanoplasmonic thin films consisting of Au nanoparticles embedded in a TiO_2_ dielectric matrix were deposited by reactive DC magnetron sputtering, an environmentally sound technology. Post-deposition treatments, such as annealing and Ar plasma, allowed the development of the sensing platform based on Au-TiO_2_ thin films. The annealing at 400 °C promoted the growth and coalescence of the Au nanoparticles dispersed throughout the TiO_2_ matrix, leading to the appearance of the LSPR band. An Ar plasma treatment was used to remove weakly bound contamination layers. Furthermore, the O_2_ plasma increased the hydrophilic surface properties, enhancing the immobilization of streptavidin on the thin film. The specific binding of biotin–HRP to streptavidin was detected by the activity of the HRP, using a protocol based on ELISA. The performance of the biosensor, consisting of immobilized-streptavidin on Au-TiO_2_ thin films, was corroborated by an LSPR wavelength peak shift of 0.8 ± 0.1 nm in the presence of the analyte.

The obtained results are promising; however, more improvements are needed before this biosensor can be employed in real-life applications. Future improvements include (1) optimization of the sensitivity (RIS) of the Au-TiO_2_ thin films, by adjusting both deposition and post-deposition treatment parameters, and (2) evaluation of concentration dependency, selectivity, specificity, and reversibility of the sensing platform.

From the detection of HRP activity and LSPR sensing, it can be concluded that the functionalization protocol was successful and streptavidin-immobilized Au-TiO_2_ thin films can be further exploited as LSPR biosensors.

## Data Availability

The data presented in this study are available on request from the corresponding author.
